# Global gene expression profiling and antibiotic susceptibility after repeated exposure to the carbon monoxide-releasing molecule-2 (CORM-2) in multidrug-resistant ESBL-producing uropathogenic *Escherichia coli*

**DOI:** 10.1371/journal.pone.0178541

**Published:** 2017-06-07

**Authors:** Charlotte Sahlberg Bang, Isak Demirel, Robert Kruse, Katarina Persson

**Affiliations:** School of Medical Sciences, Faculty of Medicine and Health, iRiSC—Inflammatory Response and Infection Susceptibility Centre, Örebro University, Örebro, Sweden; Universidade Nova de Lisboa Instituto de Tecnologia Quimica e Biologica, PORTUGAL

## Abstract

Treatment of urinary tract infections is today a challenge due to the increasing prevalence of multidrug-resistant ESBL-producing uropathogenic *Escherichia coli* (UPEC). There is an urgent need for new treatment strategies for multidrug-resistant UPEC and preferably with targets that have low potential for development of resistance. Carbon monoxide-releasing molecules (CORMs) are novel and potent antibacterial agents. The present study examines the transcriptomic targets of CORM-2 in a multidrug-resistant ESBL-producing UPEC isolate in response to a single exposure to CORM-2 and after repeated exposure to CORM-2. The bacterial viability and minimal inhibitory concentration (MIC) were also examined after repeated exposure to CORM-2. Microarray analysis revealed that a wide range of processes were affected by CORM-2, including a general trend of down-regulation in energy metabolism and biosynthesis pathways and up-regulation of the SOS response and DNA repair. Several genes involved in virulence (*ibpB)*, antibiotic resistance *(marAB*, *mdtABC*) and biofilm formation (*bhsA*, *yfgF*) were up-regulated, while some genes involved in virulence *(kpsC*, *fepCEG*, *entABE)*, antibiotic resistance (*evgA*) and biofilm formation (*artIP*) were down-regulated. Repeated exposure to CORM-2 did not alter the gene expression patterns, the growth inhibitory response to CORM-2 or the MIC values for CORM-2, cefotaxime, ciprofloxacin and trimethoprim. This study identifies several enriched gene ontologies, modified pathways and single genes that are targeted by CORM-2 in a multidrug-resistant UPEC isolate. Repeated exposure to CORM-2 did not change the gene expression patterns or fold changes and the susceptibility to CORM-2 remained after repeated exposure.

## Introduction

Nearly one-fifth of all uropathogenic strains of *E*. *coli* (UPEC) are resistant to the most commonly used antibiotics [1]. Therapeutic options are limited for extended spectrum beta-lactamase (ESBL)-producing *E*. *coli*, where the bacteria have acquired a plasmid with genes that code for the enzyme ESBL. ESBL-producing *Enterobacteriaceae* spp. contain genes that code for the ESBL enzyme, and several different ESBL enzyme variants (TEM, SHV, CTX-M) have been identified. ESBL-producing *E*. *coli* can inactivate most of the beta-lactam antibiotics and cephalosporins and frequently demonstrate co-resistance to other antibiotics, such as aminoglycosides and quinolones [2]. The most significant factor for the development of antimicrobial resistance has been found to be selection pressure caused by antibiotics [3]. In Europe, an association between use of antimicrobial drugs and occurrence of resistance has been described at a country level [4]. Development of resistance may arise after mutations through stable genetic alterations or be an adaptive phenomenon characterised by induced tolerance when the drug is present [5]. Mechanisms of antibiotic resistance include enzymatic modification of the antibiotic, reprogramming or camouflaging the target by mutation and efflux pumps which pump the antibiotic out of the cell [6].

Carbon monoxide (CO) has been ascribed a novel role as a host defence molecule with bactericidal effects [7]. CO is produced endogenously as a result of heme metabolism through the enzyme heme oxygenase (HO) and acts as a potent regulatory and protective molecule with e.g., anti-apoptotic, anti-inflammatory and anti-proliferative effects [8]. Metal carbonyl compounds or CO-releasing molecules, CORMs, for temporal and spatial CO-delivery have been developed for therapeutic applications [9]. CO easily diffuses through membranes, while CO derived from metal carbonyl compounds may be internalized into bacteria through a Trojan horse mechanism [10], [11]. The effect of CORMs on non-pathogenic *E*. *coli* seems extensive, including actions on heme-containing proteins, and a wide range of transcriptional modifications in key metabolic pathways have been observed by CORMs [11], [12], [13], [14]. A synergistic effect of CO and the metal ion co-ligand in CORMs seems to be required for full bactericidal effect [14], [15]. Our previous results show that CORM-2 has bactericidal effects against multidrug-resistant ESBL-producing UPEC [16].

There is an urgent need for new treatment strategies suitable for targeting bacteria that are resistant to traditional antibiotics. One strategy for overcoming resistance may be to develop inhibitors of novel targets, assuming that new chemical entities are not susceptible to existing resistance mechanisms [17]. Interestingly, CORMs may be less likely to cause development of resistance mechanisms, due to multiple and different targets than existing antibiotics [9]. One of the few known carbon monoxide resistance genes is *cor*, which counteracts CO toxicity in *Mycobacterium tuberculosis* [18]. In addition, deletion of genes implicated in the process of biofilm formation (*tqsA* and *bhsA)* results in higher resistance to CORM-2 in non-pathogenic *E*. *coli*, while strains mutated in methionine related genes are hypersensitive to CORM-2 [12]. Gene profiling studies on CORMs have up to now only been carried out in non-pathogenic *E*. *coli* K12 strains [11], [12], [13], [14]. The effects of CORMs on gene expression in pathogenic bacteria, such as UPEC strains, are therefore unknown. Moreover, studies addressing the potential for bacteria to develop resistance to CORMs have not yet been performed.

The aim of the present study was to use global gene profiling to assess the transcriptomic impact of CORM-2 in a multidrug-resistant ESBL-producing UPEC isolate. In addition, possible changes in gene expression, antibiotic susceptibility and virulence properties were evaluated after repeated exposure to CORM-2.

## Materials and methods

### Reagents

CORM-2 (tricarbonyldichlororuthenium (II) dimer ([Ru(CO)_3_Cl_2_]_2_)) (Sigma-Aldrich, St. Louis, MO, USA) and trimethoprim (Sigma-Aldrich) were prepared by dissolution in dimethyl sulfoxide (DMSO). Cefotaxime and ciprofloxacin (Sigma-Aldrich) were prepared in sterile water. All reagents were freshly prepared or used from stock solutions.

### Bacterial strains

Two clinical UPEC strains, the ESBL-producing *E*. *coli* isolate 7 (ESBL7) and the non-ESBL-producing isolate UPEC2, were subjected to primary susceptibility testing through the disk diffusion method at the Department of Laboratory Medicine, Microbiology, Örebro University Hospital. ESBL7 was confirmed as ESBL-producing by detecting clavulanic acid reversible resistance for oxyiminocephalosporins and found to belong to the CTX-M-15 enzyme type and sequence type 131 [19]. ESBL7 showed resistance to cefotaxime (CTX), ceftazidime (CAZ), trimethoprim (TMP), ciprofloxacin (CIP) and mecillinam (MEL). UPEC2 was susceptible to CTX, CIP, MEL, TMP and nitrofurantoin (NIT). The commensal *E*. *coli* K12 strain MG1655 was used from laboratory stocks. The study did not involve analysis of human data, specimens or tissue samples.

### Bacterial media and growth conditions

Cultures were maintained on tryptic soy agar (TSA) (Becton Dickinson, Le Pont Claix, France). Overnight cultures were grown in Difco Luria-Bertani (LB) broth (Lennox; Franklin Lakes, NJ, USA) at 37°C aerobically on a shaker at 200 rpm.

### Repeated exposures to CORM-2 or vehicle

**Fig 1:**
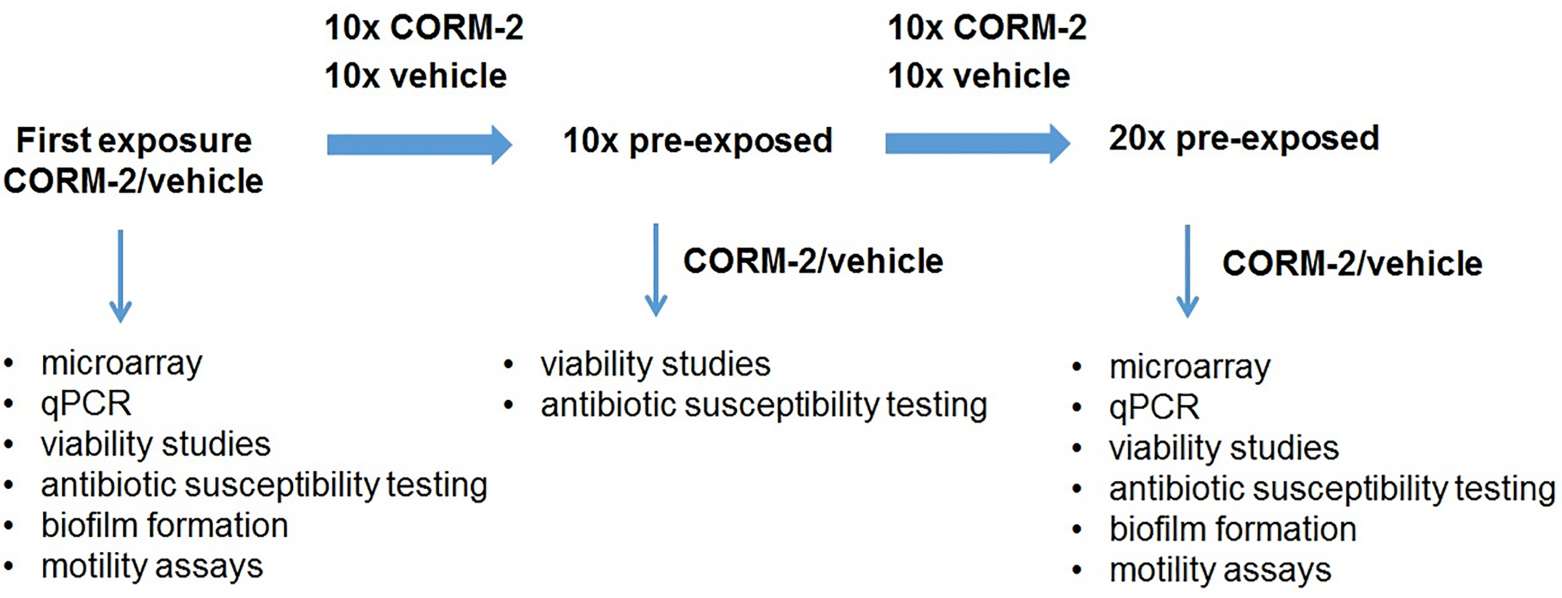
Summary of the experimental design.

Bacteria (ESBL7, UPEC2, MG1655; picked from 5–10 colonies) were suspended in 1 ml of PBS, yielding a suspension corresponding to the turbidity of McFarland 0.5, and diluted 1:100 in minimal salt (MS)-medium (~10^6^ CFU/ml). MS-medium was prepared as previously described [20] (1.3% [wt/vol] Na_2_HPO_4_, 0.3% KH_2_PO_4_, 0.05% NaCl, and 0.1% NH_4_Cl supplemented with 20 mM glucose, 2 mM MgSO_4_, 100 μM CaCl_2_, and 0.25% Casamino Acids). The suspension was exposed to CORM-2 (250 μM) or vehicle (2.5% DMSO) for 4 hours at 37°C. A volume (10 μl) was spread onto TSA-agar plates and incubated at 37°C overnight. This procedure was repeated 10 times (10x, ~45 generations) or 20 times (20x, ~90 generations). For experimental design, see **Fig 1**.

### RNA isolation

Overnight cultures of ESBL7 from the original isolate, or isolates pre-exposed 20 times to CORM-2 or vehicle, were used to inoculate MS-medium to an optical density (OD_620_) of 0.1, followed by exposure to CORM-2 (250 μM) or vehicle (2.5% DMSO) for 30 min at 37°C. RNA isolation was performed using an RNeasy mini kit (Qiagen Technologies, Hilden, Germany), according to the manufacturer’s protocol. DNA decontamination treatment was performed using Turbo DNase (Qiagen) and the quantity and purity of the purified RNA samples were determined using a spectrophotometer Nanodrop-1000 (Nanodrop Technologies Inc., Wilmington, DE, USA) by measuring the absorbance (*A*_260, 230, 280_) and calculating absorbance ratios (*A*_260_/*A*_230_ and *A*_260_/*A*_280_)_._ All samples had *A*_260_/*A*_230_ and *A*_260_/*A*_280_ ratios above 1.9. The RNA integrity was analysed using Agilent 2100 Bioanalyzer (Agilent Technologies, Palo Alto, CA, USA) in conjunction with RNA 6000 Nano LabChip kit (Agilent Technologies) according to the manufacturer’s protocol. RNA integrity number (RIN) values were > 8.5 for all samples.

### Microarray analysis

High-quality total RNA was used to prepare labelled cRNA with One-color Low Input Quick Amp WT Labelling Kit (Agilent) according to the manufacturer’s instructions. The cDNA synthesis was performed by using WT Primer Mix and cDNA Master Mix (Agilent). Labelled samples were hybridised onto G4813A *E*. *coli* gene expression Microarray 8×15K glass slides (Agilent) containing 15 208 *E*. *coli* probes. Microarrays were scanned with a G2565 CA array laser scanner (Agilent) followed by image analysis and data extraction with Feature Extraction Software version 10.7.3.1 (Agilent). Four experimental groups with 4 biological replicates in each group were analysed (total of 16 RNA samples).

### Quantitative real-time PCR (qPCR)

cDNA synthesis (0.1 μg of total RNA) was performed by using High Capacity cDNA Reverse Transcription Kit for single-stranded cDNA synthesis (Applied Biosystems, CA, USA) according to manufacturer`s protocol. qPCR was performed with Maxima SYBR Green qPCR Master Mix (ThermoFisher Scientific, MA, USA) according to manufacturer’s instructions. 200–300 nM of primer and 5 ng template cDNA was added to each supermix. Primers were ordered from Eurofins MWG Synthesis GmbH (Ebersberg, Munich, Germany) (**S1 Table**). The RT-PCR amplification was performed in a CFX96 Touch™ Real-Time PCR Detection System (Biorad, CA, USA) using the following protocol: initial denaturation at 95°C for 10 min, 40 cycles of denaturation at 95°C for 15 s followed by annealing at 60°C for 30 s and extension at 72°C for 30 s. Each PCR was followed by a dissociation curve analysis between 60–95°C. The Ct values were analysed by the comparative Ct (ΔΔCt) method and normalized to the endogenous control gapA (encoding glyceraldehyde 3-phosphate dehydrogenase A). Fold difference was calculated as 2^-ΔΔCt^.

### Determination of bacterial viability after exposure to CORM-2

Overnight culture grown in LB broth was diluted 1/1000 in MS-medium (to ~10^6^ CFU/ml) and further incubated at 37°C on a shaker at 200 rpm to early log phase (OD_620_ = 0.1). The bacterial concentrations of the initial inocula used in these experiments were in the range of 10^7^−10^8^ CFU/ml. Thereafter, the bacteria were exposed to CORM-2 (250–500 μM) and grown for up to 24 h in darkness at 37°C. Time-zero samples (starting inocula) were taken and the number of viable colonies determined as described below. Samples were taken at different times after addition of CORM-2 (1, 2, 4, 8 and 24 h) depending on the experimental protocol. All samples were diluted in PBS and at least three serial dilutions were plated on TSA-plates. Following overnight culture at 37°C, bacterial CFU/ml was determined as the mean of two dilutions. Viability was calculated as the CFU/ml in CORM-2 exposed cultures divided by the number of CFU/ml formed upon plating of the initial starting inocula and expressed as log CFU/ml.

### Determination of minimum inhibitory concentration (MIC)

MIC (minimum inhibitory concentration) for CORM-2, cefotaxime, ciprofloxacin and trimetophrim was determined using the broth dilution test. The test substances were inoculated with a bacterial suspension (~10^6^ CFU/ml) in LB-broth or MS-medium (CORM-2) on 96-well plates for 18–20 h at 37°C. All MIC tests were performed in duplicate and at least twice. The MIC was read as the lowest concentration yielding no visible growth.

### Analysis of biofilm formation

Overnight cultures in LB-broth were used to inoculate (at 0.1%) fresh MS-medium to an OD_620_ of approximately 0.05. The bacteria were seeded into 96-well plastic plates (Nunc C96 Microwell plate, Nunc A/S, Roskilde, Denmark) and exposed to CORM-2 (250 μM) or vehicle (2.5% DMSO). After 24 h of incubation under static conditions at 37°C, biofilm formation was quantified by the crystal violet method as previously described [12]. The absorbance at 540 nm was measured by spectrophotometer (Multiscan Ascent, Thermo Labsystems, Helsingfors, Finland). The experiments were repeated three times in quadruplicate.

### Motility assays

Overnight cultures in LB-broth were used to inoculate (at 0.1%) fresh MS-medium to an OD_620_ of approximately 0.1, followed by exposure to 250 μM CORM-2 or vehicle (2.5% DMSO). Swimming motility plates (0.3% agar) and swarming motility plates (0.5% agar) were prepared as previously described [21] and bacterial suspensions were inoculated on the plates. One μl of bacterial suspension was stabbed into the swimming agar plates and 5 μl bacterial suspension spotted on swarming agar plates. The distance of migration (the diameter of the growth around the inoculation site) was measured after incubation for 14 h (swimming plates) or 20 h (swarming plates) at 37°C. The experiments were repeated three times in duplicate.

### Host renal cell activation

The human renal epithelial cell line A498 (HTB-44) was obtained from American Type Culture Collection (Manassas, USA) and cultured in Dulbecco's modified eagle medium (DMEM, Sigma-Aldrich) containing 10% fetal bovine serum (FBS), 2 mM L-glutamine, 1 mM non-essential amino acids (all from Invitrogen Ltd, Paisley, UK) at 37°C in a 5% CO_2_ atmosphere. During experiments, the FBS concentration was reduced to 2%. The A498 epithelial cells were stimulated with overnight cultures of ESBL7 representing the original isolate, or isolates pre-exposed 20 times to CORM-2 or vehicle. A multiplicity of infection (MOI) of 10 was used. Cell supernatants were collected after stimulation for 6 h and centrifuged for 5 min at 5000 x g and stored at– 80°C. IL-6 and IL-8 cytokine production were measured using human IL-8 and IL-6 kits (ELISA MAX™ Deluxe Sets, BioLegend, San Diego, CA, USA) according to manufacturer's protocol and measured on a spectrophotometer (Multiscan Ascent). Cell cytotoxicity was determined using the Pierce™ LDH Cytotoxicity Assay Kit (TermoFisher Scientific, MA, USA) and absorbance measured on a spectrophotometer (Multiscan Ascent). Samples were normalized to unstimulated and lysed control cells.

### Statistical analysis and microarray data processing

Data are expressed as mean ± SEM. Student’s t-test was used to compare two groups and a one-way analysis of variance (ANOVA) parametric test was used for comparison of multiple groups, followed by Bonferroni multiple testing correction using the software GraphPad Prism (GraphPad Software Inc., La Jolla, CA, USA). Results were considered statistically significant at p-values < 0.05. Microarray data analysis was performed using GeneSpring GX version 12.1 (Agilent) after per chip and 75^th^ percentile shift gene normalization of samples. Statistical significant entities were obtained using the one-way ANOVA parametric test, followed by Tukey HSD post hoc test and Bonferroni FWER multiple testing correction, with a statistical significance set at a corrected p-value < 0.05 and a biological significance set at a fold change ≥ 2. Significant GO term enrichment and single experiment pathway analysis (SEA), was set at a p-value < 0.05 and < 0.1, respectively. n = number of independent experiments.

Genes that exhibited a two-fold or higher increase or decrease (p < 0.05) were further classified by use of gene annotations in NCBI http://www.ncbi.nlm.nih.gov, EcoCyc http://ecocyc.org and literature mining. In addition, a virulence factor list for *E*. *coli* was generated through the PATRIC database (www.patricbrc.org), MESH virulence term association and literature mining. Gene expression data is available in the GEO database with the accession number GSE87627.

## Results

### Analysis of transcriptional alterations in response to CORM-2

**Fig 2:**
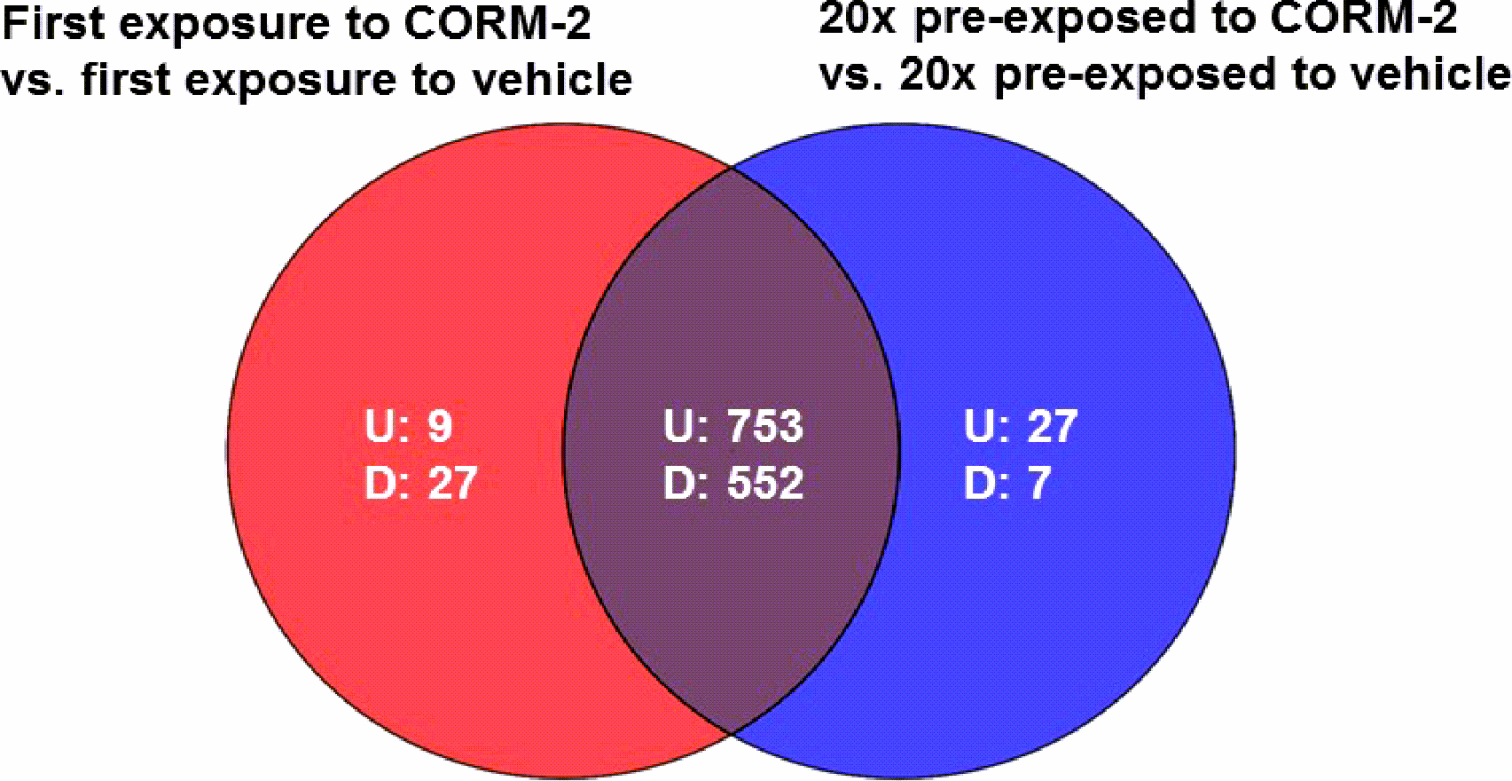
Venn diagram of differently expressed entities in ESBL isolate 7. Shown in red, first-time exposure to CORM-2 (250 μM) versus first-time exposure to vehicle (2.5% DMSO); in blue, pre-exposed 20 times to CORM-2 versus pre-exposed 20 times to vehicle. Up- and down-regulated entities are designated U and D respectively (n = 4 in each group). Overlapping regions represent entities present in both experimental conditions.

Microarray analysis was performed to analyse the gene expression alterations of ESBL7 in response to first-time exposure to CORM-2 and after pre-exposure 20 times to CORM-2. A total of 1 305 entities, common for both experimental settings, were differentially expressed with at least a two-fold change compared with vehicle (**Fig 2).** Of the 1 305 entities with altered transcription, 753 entities were up-regulated and 552 entities were down-regulated. Some differentially expressed gene entities were not shared between the experimental settings and were only found in response to first-time exposure to CORM-2 or after pre-exposure 20 times to CORM-2. Specific alterations in gene expression in response to first-time exposure and after exposure 20 times to CORM-2 showed 9 and 27 up-regulated and 27 and 7 down-regulated entities, respectively (**Fig 2**).

### Gene ontology analysis

Gene ontology (GO) analysis were performed on gene entities for each of the experimental settings. In total, 9 gene ontologies were enriched by the differentially expressed entities (**Table 1**). The enriched gene ontologies were common and found both in response to first-time exposure to CORM-2 and after pre-exposure 20 times to CORM-2. The enriched gene ontology classes were cell communication, SOS response, cellular response to external stimulus, cellular response to extracellular stimulus, response to extracellular stimulus, fermentation, cellular response to DNA damage stimulus, DNA repair and cellular response to stress (**Table 1**). The differentially expressed genes enriching the different gene ontology classes are summarized in **Table 2**and **S2 Table**.

**Table 1:** Enriched gene ontologies, common for both first-time exposed and 20 times repeated exposure to CORM-2 (250 μM) versus vehicle (2.5% DMSO) in ESBL-producing *E*. *coli*.

GO ID	GO term	Adjusted p-value	Count in selection	Count in total	No of genes up-/down-regulated
7154	cell communication	0.000	16	24	+16
9432	SOS response	0.000	16	24	+16
71496	cellular response to external stimulus	0.000	16	24	+16
31668	cellular response to extracellular stimulus	0.000	16	24	+16
9991	response to extracellular stimulus	0.000	22	41	+18/-4
6113	fermentation	0.005	17	33	+6/-11
6974	cellular response to DNA damage stimulus	0.01	20	45	+17/-3
6281	DNA repair	0.01	20	45	+17/-3
33554	cellular response to stress	0.01	22	52	+19/-3

**Table 2:** Differentially expressed genes of ESBL-producing *E*. *coli* following exposure to CORM-2 (250 μM) versus vehicle (2.5% DMSO).

Gene	Fold change	Fold change	Gene product
symbol	First exposure	20x pre-exposed	
	CORM-2 vs	CORM-2 vs	
	first exposure	20x pre-exposed	
	vehicle	vehicle	
**Represented in all enriched ontologies**			
*polB*	22.5	25.6	DNA polymerase II
*sulA*	13.9	15.6	suppressor of lon; inhibits cell division and ftsZ ring formation
*recA*^*a*^	13.6	14.2	DNA strand exchange and renaturation, DNA-dependent ATPase
*yebG*	12.5	15.3	DNA damage-inducible protein regulated by LexA
*dinI*	11.8	17.6	damage-inducible protein I
*recN*	11.4	13.0	protein used in recombination and DNA repair
*umuD*	10.2	14.5	SOS mutagenesis; error-prone repair
*umuC*	9.1	10.3	SOS mutagenesis and repair
*lexA*	8.2	10.8	regulator for SOS
*ruvB*	3.8	3.8	Holliday junction helicase subunit A
*uvrD*	3.8	2.9	DNA-dependent ATPase I and helicase II
*ruvA*	2.5	3.3	Holliday junction helicase subunit B
*uvrA*	2.6	2.5	excision nuclease subunit A
*uvrB*	2.1	2.4	DNA repair; excision nuclease subunit B
**Represented only in cell communication, SOS response, cellular response to external stimulus, cellular response to extracellular stimulus, response to extracellular stimulus, DNA repair or cellular response to stress**			
*ydjM*	21.3	18.2	inner membrane protein regulated by LexA
*dinB*	5.2	6.1	damage-inducible protein P; putative tRNA synthetase
**Represented only in cellular response to DNA damage stimulus**			
*mutM*	25.2	25.8	formamidopyrimidine/5-formyluracil/ 5-hydroxymethyluracil DNA glycosylase
*recF*	7.5	5.7	ssDNA and dsDNA binding, ATP binding
*mug*	3.2	3.5	G/U mismatch-specific DNA glycosylase
*phr*	-3.0	-2.3	deoxyribodipyrimidine photolyase
*alkB*	-2.9	-2.6	DNA repair system specific for alkylated DNA
**Represented only in cellular response to DNA damage stimulus**			
*ung*	-2.6	-2.0	uracil-DNA-glycosylase
**Represented only in response to extracellular stimulus**			
*sspB*	2.9	2.4	stringent starvation protein B
*sspA*	2.3	2.0	regulator of transcription; stringent starvation protein A
*yjiY*	-22.9	-19.2	putative carbon starvation protein
*slp*	-8.2	-7.1	outer membrane protein induced after carbon starvation
*psiF*	-3.3	-2.3	induced by phosphate starvation
*rspB*	-2.9	-2.7	starvation sensing protein

Presented genes are derived from significant enrichment in the gene ontologies cell communication, SOS response, cellular response to external stimulus, cellular response to extracellular stimulus, response to extracellular stimulus, cellular response to DNA damage stimulus, DNA repair or cellular response to stress. n = 4

^a ^also represented in virulence

### Pathway analysis

Single experiment pathway analysis (SEA) was performed in order to discover affected pathways and to further categorize the altered gene entities according to biological function. A total of 15 pathways were enriched and all were related to metabolism. Fourteen of these affected pathways were common and found both in response to first-time exposure to CORM-2 and after pre-exposure 20 times to CORM-2 (**Table 3**). The pathway carnitine degradation I was enriched only in response to first-time exposure to CORM-2.

**Table 3:** Single experiment pathway analysis of ESBL-producing *E*. *coli* gene entities.

Fold change				
Common in CORM-2 vs vehicle				
Pathway	p-value	Matched	Pathway	No of genes
		entities	entities	up-/down-regulated
glycolysis I (from glucose-6P)	0.045	2	16	-2
glycolysis II (from fructose-6P)	0.045	2	3	-2
gluconeogenesis I	0.083	2	14	-2
glucose and xylose degradation	0.003	4	6	-3/+1
mixed acid fermentation	0.002	3	3	-1/+1^a^
superpathway of N-acetylneuraminate degradation	0.003	4	6	-4
superpathway of 5-aminoimidazole ribonucleotide biosynthesis	0.083	2	4	-2
superpathway of chorismate metabolism	0.079	6	24	-5/+1
superpathway of histidine, purine and pyrimidine biosynthesis	0.043	4	12	-4
superpathway of lysine degradation	0.017	2	11	-2
superpathway of phenylalanine, tyrosine and tryptophan biosynthesis	0.050	3	7	-3
superpathway of pyrimidine deoxyribonucleotides de novo biosynthesis	0.045	2	15	-1/+1
superpathway of tryptophan biosynthesis	0.050	3	7	-3
tryptophan biosynthesis	0.002	3	9	-3

Presented pathways are affected following exposure to CORM-2 (250 μM) versus vehicle (2.5% DMSO).

^a^ part of protein complex

### Alterations in gene expression common for first-time exposure to CORM-2 and pre-exposure 20 times to CORM-2

A more detailed study of the alterations in expression of virulence, antibiotic resistance and biofilm genes was performed. Some genes involved in virulence were induced following exposure to CORM-2 (such as *ibpB*, *recA*, *ycfQ*), but many genes were repressed (such as *kpsC*, *ompW*, *ompT*, *fepEG*) (**Table 4**). Several antibiotic resistance-associated genes, such as genes coding for different multidrug efflux systems, were induced following exposure to CORM-2 (such as *mdtABC*, *marAB*, *acrD*) and some were repressed (such as *evgA*, *mdtE)* (**Table 5**). Some genes involved in biofilm formation, such as *bhsA* and *yfgF* encoding the anaerobic cyclic-di-GMP phosphodiesterase, were induced and some biofilm genes were repressed (**Table 6**). Genes involved in defence, stress response or repair, such as the gene encoding the heat shock chaperone *ibpAB* were markedly induced following exposure to CORM-2, while *hdeA* and *evgA* were repressed (**Table 7**). Three genes, *hdeA*, *cusF* **(Table 7)** and *cusX* (-58.2 first exposure; -26.3 pre-exposure 20 times) showed a significantly lower repression after pre-exposure 20 times to CORM-2 compared to first-time exposure. Differentially expressed genes associated with fimbriae and flagella are shown in **S3 Table.** CORM-2 is known to affect respiration and the majority of the differentially expressed genes involved in regulation of respiration were down-regulated (**S4 Table**).

**Table 4:** ESBL-producing *E*. *coli* genes associated with the functional category virulence that are differentially expressed following exposure to CORM-2 (250 μM) versus vehicle (2.5% DMSO).

Gene	Fold change	Fold change	Gene product
symbol	First exposure	20x pre-exposed	
	CORM-2 vs	CORM-2 vs	
	first exposure	20x pre-exposed	
	vehicle	vehicle	
*ibpB*^*a*^	2920.4	2409.3	heat shock protein
*recA*^*b*^	13.6	14.2	DNA strand exchange and renaturation, DNA-dependent ATPase
*ycfQ*	9.9	10.6	repressor for bhsA
*degP*	8.2	6.2	periplasmic serine protease Do; heat shock protein HtrA
*oxyR*	7.5	8.8	activator, hydrogen peroxide-inducible genes
*flu*	7.2	7.0	outer membrane fluffing protein
*rdoA*	6.6	6.6	Thr/Ser kinase involved in Cpx stress response
*flhE*	4.8	3.3	flagellar protein flhE precursor
*sat*	4.7	3.3	secreted auto transporter toxin
*hfq*	4.6	5.0	host factor I for bacteriophage Q beta replication
*flhB*	2.9	3.0	putative part of export apparatus for flagellar proteins
*sbmA*	2.6	2.5	sensitivity to microcin B17, possibly envelop protein
*dsbA*	2.3	3.1	protein disulfide isomerase I
*fepE*	-18.5	-14.6	ferric enterobactin transport protein fepE
*kpsC*	-16.9	-21.7	KpsC protein
*ompW*	-16.4	-13.5	outer membrane protein W precursor
*carA*	-11.7	-22.3	carbamoyl-phosphate synthetase, glutamine
*carB*	-10.3	-10.6	carbamoyl-phosphate synthase large subunit
*ompT*	-10.1	-9.2	outer membrane protein 3b
*chuT*	-9.7	-14.1	putative periplasmic binding protein
*iucA*	-8.1	-7.3	IucA protein
*serA*	-7.7	-3.9	D-3-phosphoglycerate dehydrogenase
*iucB*	-7.1	-6.3	IucB protein
*trpB*	-6.9	-13.4	tryptophan synthase, beta protein
*pyrD*	-6.8	-10.4	dihydro-orotate dehydrogenase
*fepG*	-6.5	-11.1	ferric enterobactin transport protein
*rfaL*	-6.5	-5.6	O-antigen ligase
*iucC*	-6.3	-6.1	IucC protein
*papX*	-6.0	-8.5	PapX protein
*flhD*	-5.3	-7.5	regulator of flagellar biosynthesis
*entA*	-5.2	-6.0	2,3-dihydro-2,3-dihydroxybenzoate dehydrogenase
*fepC*	-5.1	-5.6	ATP-binding component of ferric enterobactin transport
*entB*	-5.0	-6.2	2,3-dihydro-2,3-dihydroxybenzoate synthetase
*evgS*	-4.6	-6.4	putative sensor for regulator EvgA
*entE*	-4.6	-6.2	2,3-dihydroxybenzoate-AMP ligase
*chuU*	-4.0	-4.8	putative permease of iron compound ABC transport
*chuA*	-3.1	-3.7	outer membrane heme/hemoglobin receptor
*csgE*	-3.0	-3.3	curli production assembly/transport component
*rfaP*	-2.4	-2.1	lipopolysaccharide core biosynthesis

n = 4

^a ^also represented in defence, stress response or repair, Table 7

^b ^also represented in all enriched ontologies, Table 2

**Table 5:** ESBL-producing *E*. *coli* genes associated with the functional category antibiotic resistance that are differentially expressed following exposure to CORM-2 (250 μM) versus vehicle (2.5% DMSO).

Gene	Fold change	Fold change	Gene product
symbol	First exposure	20x pre-exposed	
	CORM-2 vs	CORM-2 vs	
	first exposure	20x pre-exposed	
	vehicle	vehicle	
*marA*	43.9	31.4	multiple antibiotic resistance transcriptional regulator
*mdtA*	42.4	47.4	multidrug efflux system, subunit A
*marR*	37.8	33.1	multiple antibiotic resistance protein; repressor of mar operon
*marB*	29.5	21.0	multiple antibiotic resistance protein
*mdtB*	16.0	17.4	multidrug efflux system, subunit B
*acrD*	9.7	7.1	aminoglycoside/multidrug efflux system
*ECs1864*	6.7	5.1	multidrug-efflux transport protein
*mdtC*	5.6	8.8	multidrug efflux system, subunit C
*hslJ*	3.8	4.1	heat-inducible lipoprotein involved in novobiocin resistance
*nfsA*	3.1	3.5	nitroreductase A, modulator of drug activity A
*gyrB*	2.4	2.2	DNA gyrase subunit B, type II topoisomerase
*rcsB*	2.0	2.0	response regulator in two-component regulatory system with RcsC and YojN
*evgA*^*a*^	-38.9	-25.0	response regulator in two-component regulatory system with EvgS
*mdtE*	-5.3	-4.2	anaerobic multidrug efflux transporter
*tehB*	-2.5	-2.8	tellurite, selenium resistance protein

n = 4

^a ^also represented in defence, stress response or repair, Table 7

**Table 6:** ESBL-producing *E*. *coli* genes associated with the functional category biofilm that are differentially expressed following exposure to CORM-2 (250 μM) versus vehicle (2.5% DMSO).

Gene	Fold change	Fold change	Gene product
symbol	First exposure	20x pre-exposed	
	CORM-2 vs	CORM-2 vs	
	first exposure	20x pre-exposed	
	vehicle	vehicle	
*bhsA*	191.8	242.8	biofilm, cell surface and signalling protein
*ydeH*	93.7	100.2	diguanylate cyclase, zinc-sensing
*bssS*	19.7	31.3	biofilm regulator
*tqsA*	13.3	16.5	pheromone autoinducer 2
*ybiJ*	5.8	5.8	DUF1471 family putative periplasmic protein
*yfgF*	5.3	4.6	cyclic-di-GMP phosphodiesterase
*yfaL*	2.4	2.4	adhesin
*artP*	-5.1	-7.1	ATP-binding component of arginine transport system
*bscB*	-3.7	-3.8	regulator of cellulose synthase, cyclic di-GMP binding
*artI*	-2.9	-3.1	arginine transport system, periplasmic binding protein
*csgF*	-2.2	-2.9	curli production assembly/transport component

n = 4

**Table 7:** ESBL-producing *E*. *coli* genes associated with defence, stress response or repair that are differentially expressed following exposure to CORM-2 (250 μM) versus vehicle (2.5% DMSO).

Gene	Fold change	Fold change	Gene product
symbol	First exposure	20x pre-exposed	
	CORM-2 vs	CORM-2 vs	
	first exposure	20x pre-exposed	
	vehicle	vehicle	
*ibpB*^*a*^	2920.4	2409.3	heat shock protein
*ibpA*	1424.7	1292.7	heat shock chaperone
*spy*	138.9	197.5	periplasmic ATP-independent protein refolding chaperone
*frmB*	86.5	48.5	S-formylglutathione hydrolase
*zraP*	71.5	158.7	zinc resistance protein
*soxS*	49.5	49.4	superoxide response regulon transcriptional activator; autoregulator
*pspB*	48.5	55.2	psp operon transcription co-activator
*pspC*	48.5	52.8	psp operon transcription co-activator
*pspG*	39.9	64.8	phage shock protein G
*htpG*	36.1	36.4	protein refolding molecular co-chaperone Hsp90, heat-shock protein
*yhcN*	29.9	27.2	cadmium and peroxide resistance protein
*clpB*	29.4	34.9	protein disaggregation chaperone
*dnaK*	29.2	27.2	chaperone Hsp70; DNA biosynthesis
*dnaJ*	28.5	21.0	chaperone with DnaK; heat shock protein
*htpX*	27.8	25.7	heat shock protein, integral membrane protein
*pspA*	18.0	21.6	regulatory protein for phage-shock-protein operon
*hslU*	14.8	13.9	heat shock protein hslVU, ATPase subunit
*norR*	13.7	12.5	anaerobic nitric oxide reductase DNA-binding transcriptional activator
*iscR*	10.0	10.7	isc operon transcriptional repressor; suf operon transcriptional activator
*grpE*	8.6	11.6	heat shock protein
*hslO*	8.4	6.4	heat shock protein Hsp33
*rpoH*	8.3	10.4	RNA polymerase, sigma
*loiP*	7.9	9.0	Phe-Phe periplasmic metalloprotease, OM lipoprotein
*iscS*	7.2	4.9	putative aminotransferase
*groL*	6.9	7.6	GroEL, chaperone Hsp60, peptide-dependent ATPase
*groS*	4.6	4.4	GroES, chaperone binds to Hsp60
*norW*	3.6	4.3	NADH:flavorubredoxin oxidoreductase
*hdeA*^*b*^	-108.5	-42.6	stress response acid-resistance protein
*evgA*^*c*^	-38.9	-25.0	response regulator in two-component regulatory system with EvgS
*gadB*	-30.9	-24.5	glutamate decarboxylase B, PLP-dependent
*gadX*	-17.6	-16.4	acid resistance regulon transcriptional activator
*cusF*^*b*^	-16.6	-8.6	periplasmic copper- and silver-binding protein
*gadA*	-10.9	-8.0	glutamate decarboxylase A, PLP-dependent
*aidB*	-5.3	-4.0	DNA alkylation damage repair protein
*katE*	-4.2	-4.3	catalase HPII
*katG*	-2.2	-2.6	catalase HPI

n = 4

^a ^also represented in virulence, Table 4

^b ^significant difference between first exposure and 20x pre-exposure

^c ^also represented in antibiotic resistance, Table 5

In order to confirm the microarray results, qPCR was carried out on five genes belonging to the functional category “antibiotic resistance” (*marABR* and *mdtAB*) and the two genes (*hdeA and cusF*) that showed significant differences between first-time and 20 times repeated exposure to CORM-2. In agreement with the microarray data, a marked up-regulation of *marABR* and *mdtAB* was found by qPCR (**Table 8**). Fold changes in *mdtA* expression was significantly higher after repeated exposure than after first-time exposure based on qPCR, which was not found in the microarray analysis. The microarray data showed that *cusF* and *hdeA* were significantly less repressed in cells after repeated exposure to CORM-2. qPCR data confirmed a repression of these genes but could not confirm a statistical difference between first-time and repeated exposure to CORM-2 (**Table 8**).

**Table 8:** Quantitative real-time PCR data for ESBL-producing *E*. *coli* genes following exposure to CORM-2 (250 μM) versus vehicle (2.5% DMSO).

Gene	First exposure	20x pre- exposed	Gene product
symbol	CORM-2 vs	CORM-2 vs	
	first exposure	20x pre-exposed	
	vehicle	vehicle	
	Fold change ±	Fold change ±	
	SEM	SEM	
*marA*	26 ± 4.5	30 ± 5.9	multiple antibiotic resistance transcriptional regulator
*marB*	24 ± 4.1	24 ± 4.9	multiple antibiotic resistance protein
*marR*	40 ± 3.8	41 ± 18	multiple antibiotic resistance protein; repressor of mar operon
*mdtA*^*a*^	240 ± 21	370 ± 59	multidrug efflux system, subunit A
*mdtB*	105 ± 32	170 ± 18	multidrug efflux system, subunit B
*cusF*	-1.2 ± 1.4	-0.37 ± 0.84	periplasmic copper- and silver-binding protein
*hdeA*	-0.72 ± 1.0	0.24 ± 0.91	stress response acid-resistance protein

n = 3

^a ^significant difference between first exposure and 20x pre-exposure

### Alterations in gene expression specific for first-time exposure to CORM-2 or pre-exposure 20 times to CORM-2

Although the vast majority of the differentially expressed genes were common and found both in response to first-time exposure to CORM-2 and after pre-exposure 20 times to CORM-2, some specific changes were noted. Overall, the specific changes were modest with a fold change close to 2 (**S5 Table**).

### Bacterial viability in response to repeated exposure to CORM-2

**Fig 3:**
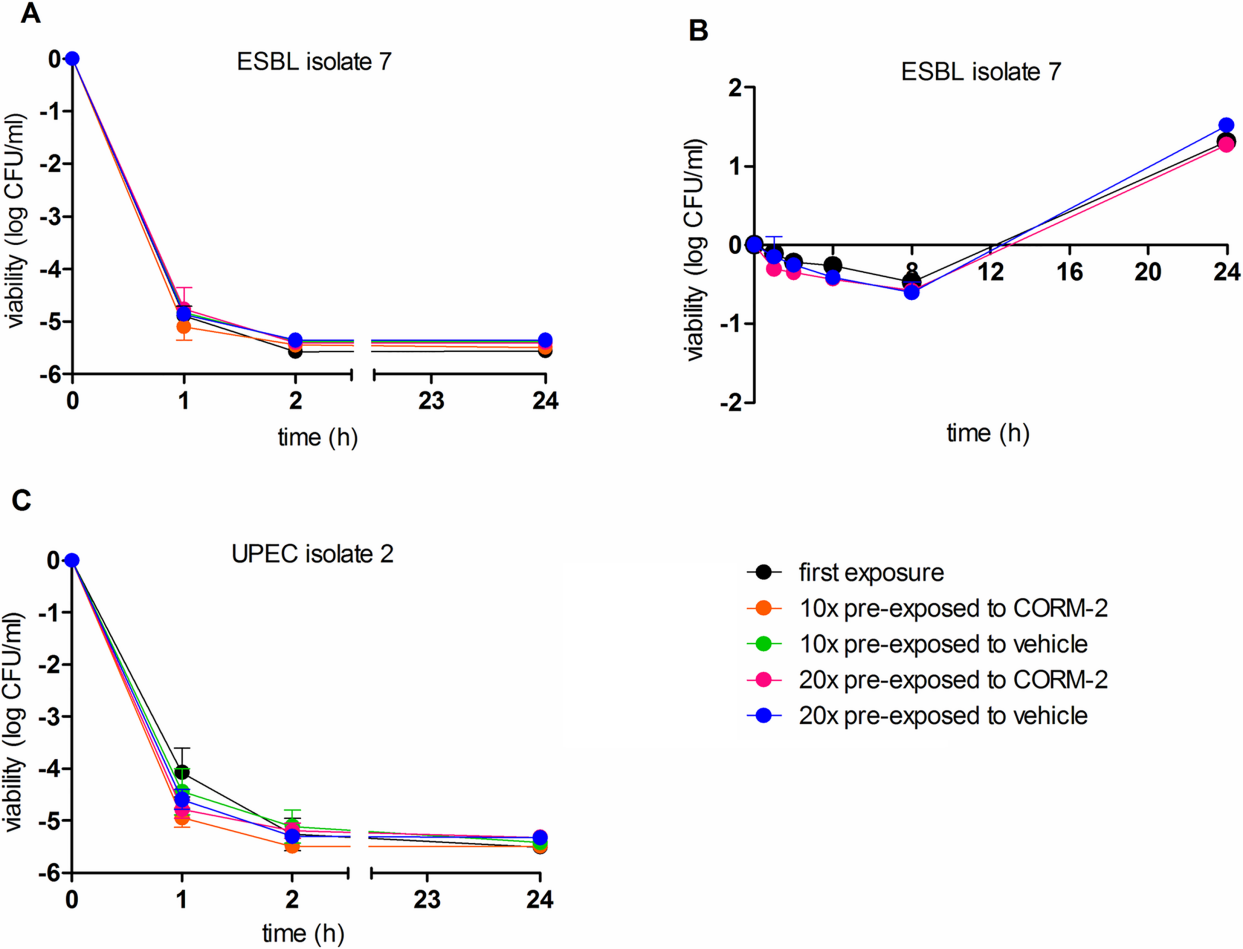
Effects of CORM-2 exposure on viability in ESBL isolate 7 and UPEC isolate 2. A) ESBL7 and C) UPEC2 were grown to early log phase in MS-medium and then exposed to CORM-2 (500 μM) for 1, 2 or 24 h. B) ESBL7 were grown to early log phase in MS-broth and then exposed to CORM-2 (250 μM) for 1, 2, 4, 8 or 24 h. Data show viability after first-time exposure and after pre-exposure 10 or 20 times to CORM-2 (250 μM) or vehicle (2.5% DMSO). Viability is presented as log CFU/ml of CORM-2 exposed bacteria compared with the initial starting inoculum. The data are shown as mean ± SEM from three independent experiments.

**Fig 4:**
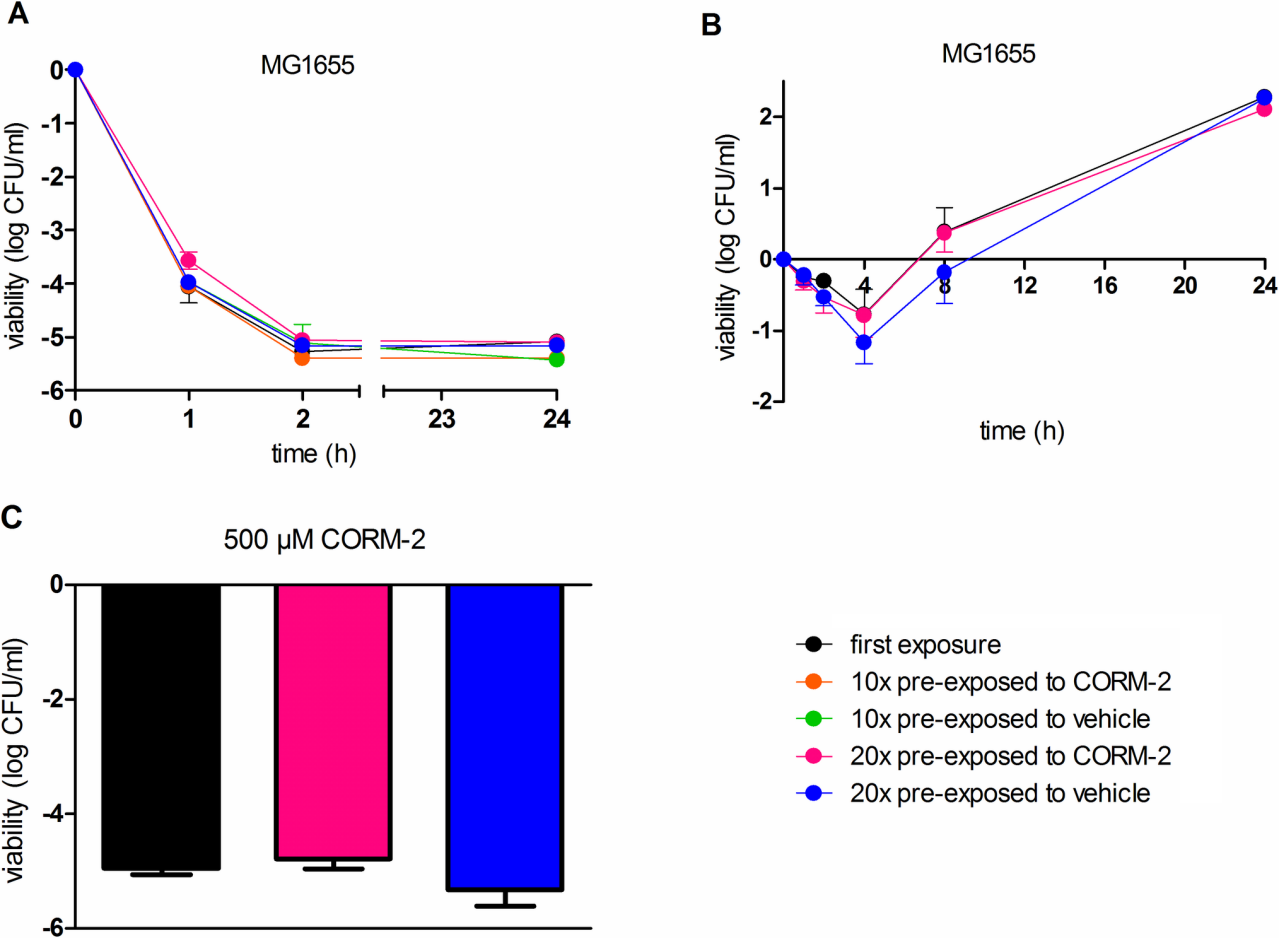
Effect of CORM-2 exposure on viability in *E*. *coli* K12 strain MG1655. MG1655 was grown to early log phase in MS-medium and then exposed to **A)** CORM-2 (500 μM) for 1, 2 or 24 h or to **B)** CORM-2 (250 μM) for 1, 2, 4, 8 or 24 h. **C)** Bacteria with a recovered growth after 24 h of exposure to 250 μM CORM-2 (*see* panel B) were re-exposed to a higher concentration of CORM-2 (500 μM) and the viability evaluated. Data show viability after first-time exposure and after pre-exposure 10 or 20 times to CORM-2 (250 μM) or vehicle (2.5% DMSO). Viability is presented as log CFU/ml of CORM-2 exposed bacteria compared with the initial starting inoculum. The data are shown as mean ± SEM from three independent experiments.

Bacterial viability studies were performed to compare the growth inhibitory effect of CORM-2 (500 μM) after first-time exposure with the inhibitory effect after pre-exposure 10 or 20 times to CORM-2 or vehicle. In the viability studies, three different bacterial isolates were used: ESBL7, a non ESBL-producing UPEC isolate (UPEC2) and a commensal *E*. *coli* K12 strain (MG1655). CORM-2 (500 μM) showed a fast bactericidal effect with a reduction of bacterial counts by 4–5 log units after 1 hour of exposure in all three isolates (**Figs 3A, 3C and 4A**). Growth inhibition peaked after 2 hours with no resumed growth during the 24-hour study period. Untreated controls showed an increased growth response of ~2 log units during the 24-hour study period (data not shown). The inhibitory effect of CORM-2 (500 μM) did not differ significantly between samples pre-exposed to CORM-2 or samples pre-exposed to vehicle. Neither were there any significant differences in response to CORM-2 (500 μM) between bacteria exposed once, 10 or 20 times to CORM-2 (**Figs 3A, 3C and 4A)**. A sub-MIC concentration of CORM-2 (250 μM) was examined in ESBL7 and MG1655, showing a bacteriostatic response for 4–8 hours with a recovered growth after 24 hours (**Figs 3B and 4B**). No significant difference in viability was found between the first-time exposure and after pre-exposure 20 times to CORM-2. To study if the recovered growth observed 24 h after exposure to 250 μM CORM-2 was caused by survival of a resistant phenotype, the bacteria were immediately re-exposed to a higher concentration of CORM-2 (500 μM). However, the sensitivity to CORM-2 (500 μM) was not reduced or dependent on the previous exposures to CORM-2 (**Fig 4C**).

### Effect of repeated exposure to CORM-2 on cefotaxime, ciprofloxacin and trimethoprim susceptibility

Determination of MIC values was performed using the broth dilution test. The MIC value for CORM-2 was determined to be 500 μM for all strains (ESBL7, UPEC2, MG1655) and MIC did not differ between first-time or repeated exposures (**Table 9**). Evaluation of MIC values was also performed to address whether repeated CORM-2 exposure affected the bacterial susceptibility to cefotaxime, ciprofloxacin and trimethoprim, antibiotics that are used to treat UTI. ESBL7 was resistant to cefotaxime and trimethoprim as expected, but the response to ciprofloxacin was indeterminate (**Table 9**). The MIC values for cefotaxime, ciprofloxacin and trimethoprim in ESBL7 did not differ between the first-time exposure and after repeated exposure to CORM-2 or vehicle. The isolates UPEC2 and MG1655 were sensitive to cefotaxime, ciprofloxacin and trimethoprim. The MIC values for cefotaxime and ciprofloxacin did not change after repeated exposure to CORM-2 in UPEC2 or MG1655 (**Table 9**). The MIC value for trimethoprim in isolate UPEC2 remained unchanged, but strain MG1655 showed a higher MIC value (1 μg/ml vs 0.5 μg/ml) for trimethoprim after pre-exposure 20 times to CORM-2 or vehicle (**Table 9**).

**Table 9:** MIC values for CORM-2, ciprofloxacin, cefotaxime and trimethoprim for ESBL-producing *E*. *coli* isolate 7, uropathogenic UPEC isolate 2 or non-pathogenic MG1655 in response to first-time exposure to CORM-2 and after 10 or 20 times pre-exposure to CORM-2 (250 μM) or vehicle (2.5% DMSO).

Antibiotic susceptibility testing, MIC values					
	First exposure CORM-2	10x CORM-2	10x vehicle	20x CORM-2	20x vehicle
**CORM-2 (μM)**					
**ESBL7**	500	500	500	500	500
**UPEC2**	500	500	500	500	500
**MG1655**	500	500	500	500	500
Breakpoint^a^ -					
**CIP (μg/ml)**					
**ESBL7**	0.5	0.5	0.5	0.5	0.5
**UPEC2**	0.031	0.031	0.031	0.031	0.031
**MG1655**	0.031	0.031	0.031	0.031	0.031
Breakpoint^a^ 0.5/1					
**CTX (μg/ml)**					
**ESBL7**	>32	>32	>32	>32	>32
**UPEC2**	0.062	0.062	0.062	0.062	0.062
**MG1655**	0.062	0.062	0.062	0.062	0.062
Breakpoint^a^ 1/2					
**TMP (μg/ml)**					
**ESBL7**	>32	>32	>32	>32	>32
**UPEC2**	0.25	0.25	0.25	0.25	0.25
**MG1655**	0.5	0.5	0.5	1	1
Breakpoint^a^ 2/4					

Abbreviations: ciprofloxacin (CIP), cefotaxime (CTX), trimethoprim (TMP)

^a ^Clinical MIC breakpoint for Enterobacteriaceae set by the SRGA and the European Committee on Antimicrobial Susceptibility Testing (EUCAST). S, susceptibility/ R, resistant. -, No clinical breakpoint.

### Effect of repeated exposure to CORM-2 on biofilm formation and motility

**Fig 5:**
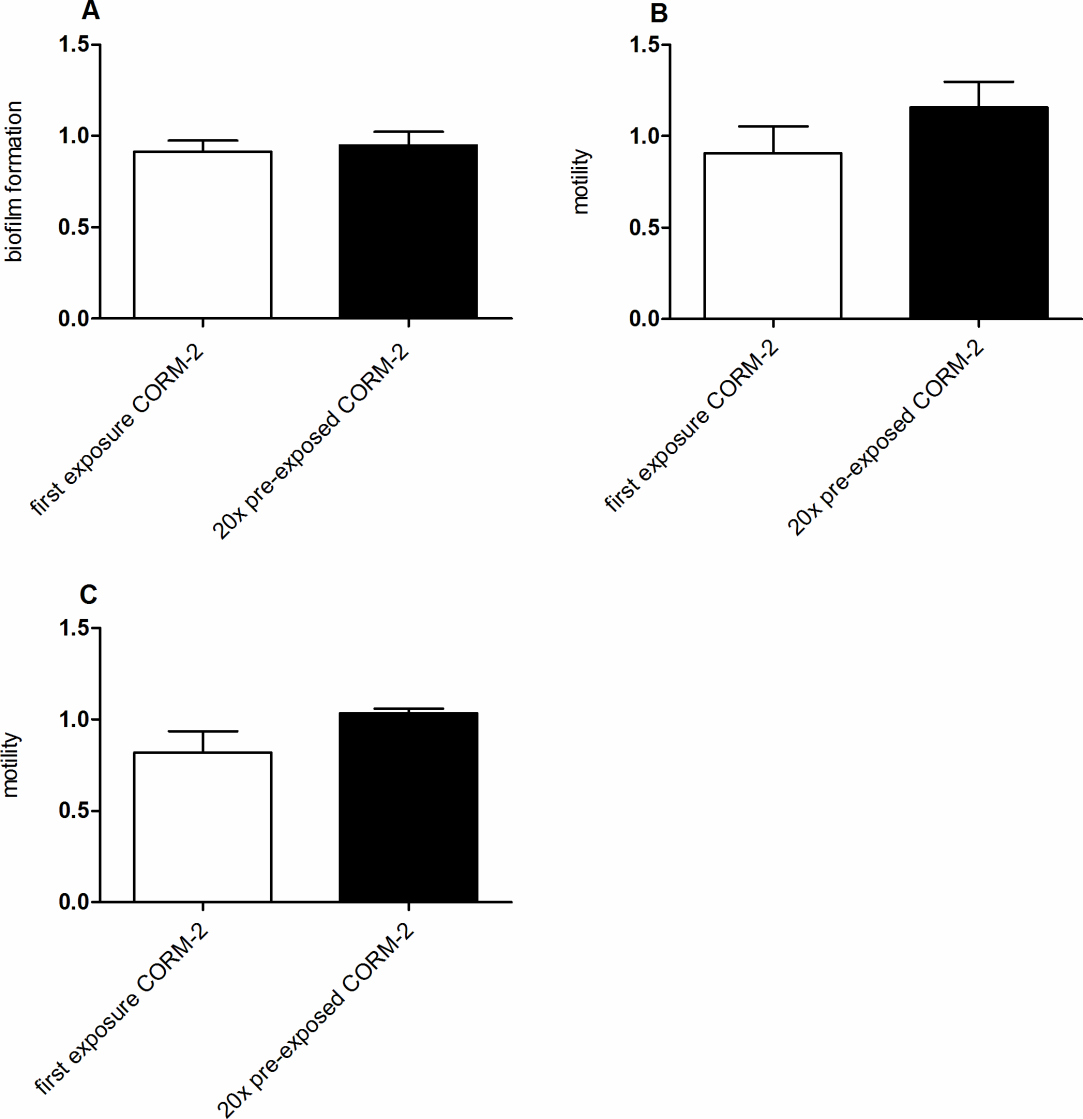
The phenotypic effect of CORM-2 on biofilm formation and motility in ESBL isolate 7. A) Biofilm formation measured after first-time exposure to CORM-2 and after pre-exposure 20 times to CORM-2 (250 μM). The biofilm formation is presented as relative changes compared to the formation evoked by the vehicle (2.5% DMSO). Motility measured on B) swimming plates and C) swarming plates after first-time exposure and after pre-exposure 20 times to CORM-2 (250 μM) or vehicle (2.5% DMSO). The motility data are presented as relative changes compared to the motility evoked by the vehicle (2.5% DMSO). Data are shown as mean ± SEM from three independent experiments.

Many genes encoding biofilm were altered in response to CORM-2 and quantification of biofilm formation was performed in ESBL7 by the crystal violet method. The basal biofilm formation in ESBL7 was low (A_540_ ~ 0.1) and the effect of CORM-2 (250 μM) on biofilm formation was minor and not significantly different from the effect evoked by the vehicle (**Fig 5A**). Neither were there any significant differences in biofilm formation between bacteria exposed once or 20 times to CORM-2 (**Fig 5A**). Several genes encoding flagella were affected by CORM-2 and to determine whether changes in expression resulted in changed motility two motility assays were performed. The swimming motility assay measures flagella driven individual cell movement and the swarming motility assay the flagella driven multicellular surface movement. ESBL7 developed the typical colonial patterns associated with swimming and swarming migration (data not shown). The effect of CORM-2 (250 μM) *per se* on motility was not significantly different from the effect evoked by the vehicle. There were no significant differences in swimming (**Fig 5B**) or swarming **(Fig 5C)** motility between bacteria exposed once or 20 times to CORM-2.

### Effect of repeated exposure to CORM-2 on host renal cell production of cytokines

**Fig 6:**
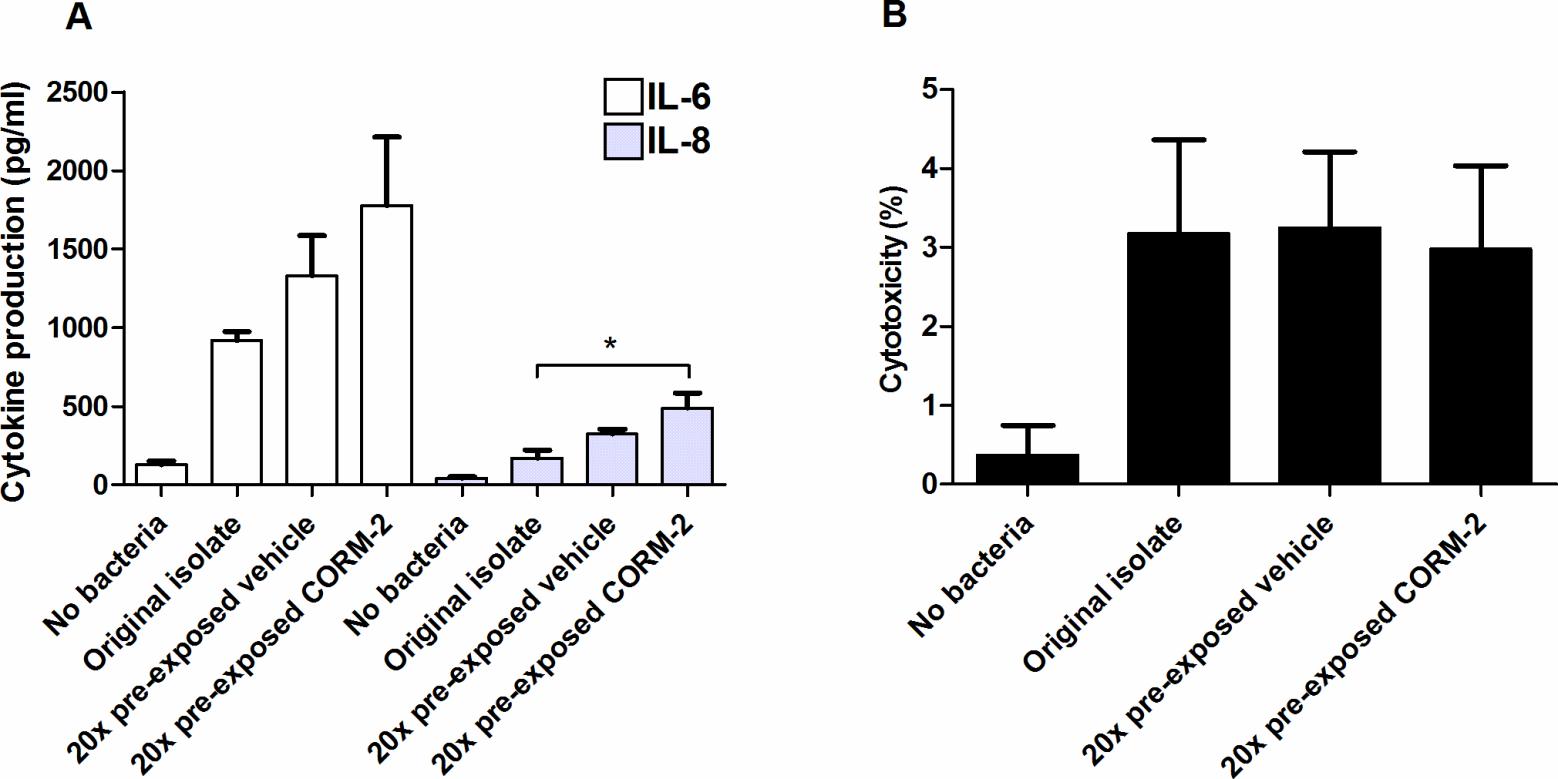
Host renal cell production of cytokines and cytotoxicity in response to ESBL isolate 7. A) IL-6 and IL-8 production from A498 renal epithelial cells after stimulation for 6 h with the original ESBL7 isolate or with ESBL7 that have been pre-exposed 20 times to CORM-2 (250 μM) or vehicle (2.5% DMSO). B) Host renal cell cytotoxicity measured as LDH-release during the same conditions as in panel A and normalized to unstimulated and lysed control cells. Data are presented as mean ± SEM from three independent experiments. Asterisk denotes statistical significance (*p<0.05).

Uroepithelial cells contribute to the initiation of host defense against UPEC through the production of various cytokines and chemokines [22]. We next addressed whether repeated CORM-2 exposure affected the ability of EBL7 to evoke IL-6 and IL-8 production from host renal epithelial cells. The original ESBL7 isolate stimulated production of IL-6 and IL-8 compared to un-stimulated A498 cells (**Fig 6A**). The cytokine production was further increased in ESBL7 pre-exposed 20 times to CORM-2 or vehicle, but only significantly higher than the original isolate for IL-8 and CORM-2 pre-exposed bacteria (**Fig 6A**). The host renal cell cytotoxicity was low (~ 3%) for all experimental conditions (**Fig 6B**).

## Discussion

Gene profiling of a multidrug-resistant ESBL-producing UPEC isolate demonstrated a significant alteration of a large number of genes after exposure to the CO-donor CORM-2. In all, close to 9% of the entities on the array were altered. However, this does not correspond to a fixed number of altered genes in the genome, since multiple entities sometimes represent the same gene. Our results are in agreement with a previous transcriptome analysis of non-pathogenic *E*. *coli* where ~9% of the total genome for anaerobically grown cells and ~4% for aerobically grown cells were altered in response to CORM-2 [12]. Thus, it appears to be an extensive flux in the transcriptome of the bacteria in order to cope with the altered environment induced by CORM-2 exposure.

The vast majority of the identified gene changes were common for bacteria exposed one time or repeatedly (20 times) to CORM-2. The enriched gene ontologies and pathway analysis stratified at the level common for both first-time exposed and repeatedly exposed samples showed that cellular responses and adaptions in metabolism genes are substantially affected. CORM-2 caused a general trend of down-regulation in energy metabolism, biosynthesis pathways, catabolism and up-regulation of the SOS response and DNA damage and repair mechanisms. A reduced fermentation is indicated by down-regulation of several genes, e.g., *frdB* and *fumB* [23], [24]. In *E*. *coli*, the reduced cofactor NADH plays a key role and without NADH reoxidation, cellular metabolism and growth are halted [25], [26]. Many genes coding for the NADH:ubiquinone oxidoreductase subunits (*nuoABCEFGHIJMN*) and trimethylamine N-oxide reductase (*torACD*) were repressed by CORM-2. A down-regulation of the nuo-operon (11 genes) by CORM-3 was also found in *E*. *coli* MG1655 [11], indicating that the NADH dehydrogenase complex is a target for CORMs. It is presumed that CO gas and CORMs will interact with quinol oxidase protein complexes of *E*. *coli* [27], but no significant changes in gene expression of *cydAB* (encoding cytochrome oxidase bd-I) or *cyoABCDE* (encoding cytochrome bo oxidase) were observed in our study. Previous transcriptomic analysis with *E*. *coli* and CORM-3, performed in defined medium under aerobic conditions, demonstrated down-regulation of *cyoABCDE* genes and a modest increase followed by a decrease in the *cydAB* genes [11], [13]. It is possible that the less defined growth conditions used in our study may explain the lack of altered expression of cytochrome genes.

There was a general trend of up-regulated genes in the enriched gene ontologies SOS response, cellular response to DNA damage stimulus, DNA repair and cellular response to stress. The SOS response is an extensive and effective response to DNA damage and the SOS response is regulated by LexA/RecA [28]. The induction of genes for *recA* and repressor *lexA* indicates an increased need for repair mechanisms in bacteria exposed to CORM-2. Many LexA regulated genes were induced (*recN*, *recA*, *sulA*, *uvrD*, *umuC*, *umuD*, *polB*), as were the damage-inducible genes *dinI* and *dinB*. DNA polymerase II, encoded by *polB*, is proposed to have a role in repair of oxidative damage and also to increase the rate of mutations during the SOS response [29]. Thus, another outcome of inducing the SOS response is increased genetic variability and the acquisition of bacterial mutations that may lead to resistance to some antibiotic drugs [30]. Moreover, bactericidal antibiotics may induce mutagenesis by stimulating the production of reactive oxygen species (ROS) [31]. Although controversial, the bactericidal cell death caused by CORM-2 may involve generation of intracellular ROS and a subsequent induction of DNA damage [32], [33].

CORM-2 has been shown to increase biofilm formation in an *E*. *coli* K12 strain [12]. BhsA is a small outer membrane protein involved in biofilm formation and stress response [34], and *bhsA* was induced ~200-fold by CORM-2 in our study. However, the overall biofilm formation, at least on plastic abiotic surface, was low in ESBL7 and the effect of CORM-2 on biofilm formation was minor. The reduced gene expression of *artP* and *artI* is in agreement with previous studies showing repression of these transport-encoding genes during biofilm formation [12], [35]. In agreement with previous results in an *E*. *coli* K12 strain [12], exposure to CORM-2 increased expression of several genes known to encode cytoplasmic adaptions and stress responses, like *ibBA*, *ibpA* and *spy* (approximately 3000-, 1500- and 140-fold increases, respectively). Notably, the fold changes of these genes were found to be considerably higher in our study. The *spy* gene is implicated in both zinc homeostasis and envelope stress responses [36], and *spy* has been identified as one of the main non-heme targets for CORM-3 [14]. Chaperone ibpA/B activities seem to promote disaggregation of protein aggregates [37], and *ibpB* has been associated with hyper-colonization of UPEC in the mouse urinary tract [38].

In the present study, a pathogenic *E*. *coli* strain was used which increases the clinical relevance of the acquired transcriptional data, including information of potential virulence genes, compared to previous studies using non-pathogenic *E*. *coli* K12 strains. Some virulence genes were up-regulated but the majority were down-regulated, like genes encoding products involved in iron transport and acquisition (*fepEG*, *entABE*). These genes have previously been reported to be up-regulated by CORM-3 exposure in an *E*. *coli* K12 strain [14]. However, there are known discrepancies when comparing transcriptomic data after CORM-2 and CORM-3 exposure and, in addition, different growth conditions, growth rates and exposure times between studies may influence the results [9]. We found a reduced expression of *kpsC* that is involved in group II capsule biosynthesis in UPEC. This gene is absent from the genome of *E*. *coli* K12 strains [39]. Interestingly, a previous study has shown that synthesis of extracellular polysaccharides, including group II capsular polysaccharide, is necessary for optimal urovirulence in the murine urinary tract [39]. Taken together, CORM-2 exposure appears to reduce the expression of many UPEC virulence factors. UPEC attachment to and/or invasion of epithelial cells are the initial steps in the pathogenesis of UTI [22]. UPEC employ multiple strategies to attenuate the initiation of the host response in order to evade the recruitment and activity of phagocytic neutrophils. Compared with commensal strains of *E*. *coli*, UPEC are able to suppress epithelial cytokine and chemokine production and many UPEC isolates elicit lower levels of IL-6 and IL-8 secretion from uroepithelial cells [40]. Our functional host renal cell experiments demonstrated an enhanced IL-8 production in response to bacteria repeatedly exposed to CORM-2, which may support the transcriptional data showing reduced expression of many virulence genes.

Overall, the alterations and fold-changes in gene expression were markedly consistent between bacteria exposed one time to CORM-2 or 20 times to CORM-2. Three genes *hdeA*, *cusF* and *cusX* were significantly less repressed after repeated exposure to CORM-2 compared with a single exposure. The *hdeA* gene encodes an HDEA protein that confers acid resistance [41] and it is possible that the intracellular pH homeostasis is altered in response to CORM-2 exposure due to impairment of the respiratory chain [20]. However, measurement of pH during bacterial growth before and during exposure to 250 μM CORM-2 for up to 4 h did not reveal any pH changes, at least not in the extracellular space (data not shown). CusCFBA is an efflux system protecting the periplasm from transition metal-mediated damage using a proton gradient [42], while the *cusX* gene encodes a hypothetical protein. qPCR analysis confirmed that *hdeA* and *cusF* were repressed by CORM-2, but no difference between single and repeated exposures was found by qPCR.

Several genes encoding efflux pump systems were altered by CORM-2. An induced expression of the genes *mdtABC* was found in agreement with a previous study performed in an *E*. *coli* K12 strain exposed to CORM-3 [14]. Further, an increased expression of *marABR* was found; *marA* is known to control expression of resistance to antibiotics like tetracycline, chloramphenicol and cephalosporins [43] and oxidative stress agents [44], by altering the expression of multiple genes on the bacterial chromosome. Consistent with microarray data, *marABR* and *mdtAB* were markedly upregulated by CORM-2 based on qPCR data. Many multidrug-resistant intestinal bacteria show an increased expression of genes for efflux pumps of the Resistance-Nodulation-cell Division (RND) family (as *acrAB-TolC*) involved in the reduction of antibiotic susceptibility [45]. In our study, the gene *acrA* was only induced in response to first-time exposure to CORM-2, while the gene *acrD* was induced both in first-time exposed and repeatedly exposed bacteria. The gene *acrD* encodes the efflux pump acrD which participates in aminoglycoside efflux [46]. However, it should be noted that the alterations in gene expression specific for first-time exposure or pre-exposure 20 times to CORM-2 were all small (approximately 2-fold), which indicates that these differences are rather uncertain and may be biological insignificant. Multidrug efflux pumps are known to confer low-level intrinsic resistance to drugs, and when mutations in regulatory genes appear, high expression levels of multidrug efflux pumps may interfere with therapeutic treatments [47]. Any new antibiotic seems to be favoured by not inducing overexpression of efflux pumps [48]. Thus, the CORM-2 evoked expression of genes for different efflux pumps may suggest a possibility for development of an antibiotic resistant phenotype. The finding that CORM-2 up-regulated genes for multidrug efflux pumps may support the notion that CORMs enter the bacteria through a specific, although yet unknown, transport mechanism [9]. However, CORMs may not *per se* activate the AcrAB-TolC multidrug efflux system since gene expression of efflux pumps is also enhanced by the SOS response [49].

The results regarding genes that encode flagella and fimbriae were not conclusive and both induced and reduced genes were noted. One of the genes exclusively induced by repeated exposure to CORM-2 was *csgC*, a gene encoding a potential curli assembly protein [50]. In previous *E*. *coli* K12 studies, an increased expression of the flagellar repressor *lrhA* was reported in response to CORM-2 [12], and expression of motility genes and functional motility was diminished in response CORM-3 [13]. RecA, one of the most up-regulated genes by CORM-2, promotes swarming motility in *E*. *coli* K12 by a yet unclear mechanism [21]. However, in our study, the changes in expression of genes responsible for flagella function did not correspond to any changes in functional cell motility, as evaluated by swimming and swarming motility assays.

Functional viability studies showed a bactericidal effect by 500 μM CORM-2 on ESBL7, the non-ESBL-producing UPEC isolate 2 and the non-pathogenic *E*. *coli* K12 strain MG1655, in agreement with previous studies [20], [16]. CORM-2, at 250 μM, caused a short-lasting growth inhibitory effect that was fully recovered after 24 h. The CORM-2-evoked growth inhibition achieved after first-time exposure was similar to the inhibition noted in samples pre-exposed 10 times or 20 times to CORM-2. Taken together, these data show that the growth inhibitory response was not attenuated after repeated exposure to CORM-2, neither in a multidrug-resistant *E*. *coli* strain nor in two antibiotic susceptible strains. In a previous study [16], we addressed whether multidrug-resistant ESBL-producing UPEC isolates were less sensitive to CORM-2 than non-pathogenic *E*. *coli* MG1655. However, no correlation between sensitivity towards CORM-2 and the pathogenic potential or antibiotic resistance of the strains was observed.

A comparison of MIC values for CORM-2 after first-time and repeated exposure to CORM-2 revealed no changes in MIC for any of the tested strains. Thus, the results obtained for CORM-2 by the antimicrobial susceptibility test supported the results from the viability studies. Increased gene expression of efflux pumps or increased mutation rates from repeated CORM-2 exposure could presumably lead to increased resistance towards other antibiotic classes. However, there were no indications of altered susceptibility (MIC values) for three classes of traditional UTI antibiotics (cefotaxime, trimethoprim or ciprofloxacin) after repeated exposure for 10 or 20 times to CORM-2. The non-pathogenic strain MG1655 showed slightly increased MIC values for trimethoprim after exposure for 20 times; however, this was also found for the DMSO vehicle. The use of DMSO in medium (0.1–10%) has been shown to cause reversion of sensitivity in *E*. *coli* strains [51] and it can therefore not be excluded that DMSO has affected the susceptibility. Moreover, an occasional two-fold difference in MIC values is expected in two-fold dilution assay. A limitation of the present study is that the applied protocol for repeated exposure to CORM-2 may not have been optimal to detect possible changes in susceptibility to CORM-2. A protocol with a gradual increase in the antibiotic concentration, starting from a very low sub-inhibitory concentration may allow sufficient time for mutations and selection [52]. In addition, extension of the experiments to include more generations may display resistance development [52]. Further studies with more extensive and different exposure protocols are certainly needed to fully evaluate development of resistance to CORMs.

## Conclusions

This is the first study addressing the potential for bacteria to develop resistance to CORMs. Repeated exposure to CORM-2 did not change the gene expression patterns or fold changes and the viability studies showed a sustained phenotypic susceptibility to CORM-2. CORM-2 caused a pronounced activation of the potentially mutagenic SOS response and an increased expression of efflux pumps that may suggest that CORM-2 has a potential for resistance development. However, CORMs seem to be favoured by interactions with multiple and novel target sites that are different from those of traditional antibiotics and pre-existing resistance mechanisms. More comprehensive studies, including sequencing of the genome and mutation analysis, are needed to evaluate the likelihood for CORMs to develop resistance. Multidrug-resistance among uropathogenic bacteria is today prominent and the risk of treatment failure is an emerging threat and a public health concern. CORMs are interesting candidate molecules for development of new antibiotics to treat UTI. In addition, adjuvant treatment strategies where CORMs are combined with established antibiotics [15] are one interesting approach that should be further evaluated.

## Supporting information

S1 Table Primers used for quantitative real-time PCR. (DOCX)

S2 Table Differentially expressed genes of ESBL-producing *E*. *coli* following exposure to CORM-2 (250 μM) versus vehicle (2.5% DMSO). Presented genes are derived from significant enrichment in the gene ontology fermentation. n = 4
(DOCX)

S3 Table ESBL-producing *E*. *coli* genes associated with fimbriae and flagella that are differentially expressed following exposure to CORM-2 (250 μM) versus vehicle (2.5% DMSO). n = 4
(DOCX)

S4 Table ESBL-producing *E*. *coli* genes associated with aerobic and anaerobic respiration that is differentially expressed following exposure to CORM-2 (250 μM) versus vehicle (2.5% DMSO). n = 4
(DOCX)

S5 Table Differentially expressed genes that were not shared and present only after first-time exposure or only after pre-exposure 20 times to CORM-2 (250 μM). n = 4
(DOCX)
