# Supplementary material for: Global gene expression profiling and antibiotic susceptibility after repeated exposure to the carbon monoxide-releasing molecule-2 (CORM-2) in multidrug-resistant ESBL-producing uropathogenic Escherichia coli
Source: PLoS One. 2017 Jun 7;12(6):e0178541. doi: 10.1371/journal.pone.0178541 (PMC5462378; doi:10.1371/journal.pone.0178541)
Supplement: S1 Table — (DOCX) [file pone.0178541.s001.docx]

**S1 Table. Primers used for quantitative real-time PCR.**

| **Gene symbol** | **Oligonucleotide sequences (5´-3´)** | **References** |
| --- | --- | --- |
| marA | *F: CATAGCATTTTGGACTGGAT* | [1] |
|  | *R: TACTTTCCTTCAGCTTTTGC* |  |
| marB | *F: ATAGCAGCTGCGCTTATTC* | [1] |
|  | *R: ACTTATCACTGCCAGTACCC* |  |
| *marR* | *F: AGCGATCTGTTCAATGAAAT* | [1] |
|  | *R: TTCAGTTCAACCGGAGTAAT* |  |
| *mdtA* | *F: TAACGCGCGCATGTTAGT* | [2] |
|  | *R: GCTATTCAGCACCCAGACAA* |  |
| *mdtB* | *F: AACTGCGTCGTCCGTTAG* | [2] |
|  | *R: GCGGTCGAACAGCAAATAAA* |  |
| *cusF* | *F: AGTCGCAATGTTCAGTCTGT* | [3] |
|  | *R: CAGCAATCGGATCGTGATGG* |  |
| *hdeA* | *F: GTCAACTCCTGGACCTGTGA* | [3] |
|  | *R: CGATAGCTGGGGTTACGGTT* |  |
| *gapA* | *F: AAGTTGGTGTTGACGTTG* | [4] |
|  | *R: AGCGCCTTTAACGAACATCG* |  |

1. Viveiros M, Dupont M, Rodrigues L, Couto I, Davin-Regli A, Martins M, et al. Antibiotic stress, genetic response and altered permeability of *E. coli*. PloS one. 2007;2(4):e365.

2. Allen HK, An R, Handelsman J, Moe LA. A response regulator from a soil metagenome enhances resistance to the beta-lactam antibiotic carbenicillin in *Escherichia coli*. PloS one. 2015;10(3):e0120094.

3. Page DC, Lander ES. Genomics at Estonian Biocentre; 2017 [cited 2017 april 20]. Database: Primer3web [Internet]. Available from: <http://primer3.ut.ee/>.

4. Alteri CJ, Mobley HL. Quantitative profile of the uropathogenic *Escherichia coli* outer membrane proteome during growth in human urine. Infect Immun. 2007;75(6):2679-88.
