# Supplementary material for: Global gene expression profiling and antibiotic susceptibility after repeated exposure to the carbon monoxide-releasing molecule-2 (CORM-2) in multidrug-resistant ESBL-producing uropathogenic Escherichia coli
Source: PLoS One. 2017 Jun 7;12(6):e0178541. doi: 10.1371/journal.pone.0178541 (PMC5462378; doi:10.1371/journal.pone.0178541)
Supplement: S2 Table — Presented genes are derived from significant enrichment in the gene ontology fermentation. n = 4 (DOCX) [file pone.0178541.s002.docx]

**S2 Table**. **Differentially expressed genes of ESBL-producing *E. coli* following exposure to CORM-2 (250 µM) versus vehicle (2.5% DMSO).**

| **Gene** | **Fold change** | **Fold change** | **Gene product** |
| --- | --- | --- | --- |
| **symbol** | **First exposure** | **20x pre-exposed** |  |
|  | **CORM-2 vs** | **CORM-2 vs** |  |
|  | **first exposure** | **20x pre-exposed** |  |
|  | **vehicle** | **vehicle** |  |

| *frmA* | 33.8 | 27.1 | alcohol dehydrogenase class III, formaldehyde dehydrogenase, glutathione-dependent |
| --- | --- | --- | --- |
| *zraS* | 6.6 | 8.0 | sensor kinase for HydG, hydrogenase 3 activity |
| *zraR* | 5.6 | 7.4 | response regulator of hydrogenase 3 activity |
| *ldhA* | 4.5 | 3.7 | fermentative D-lactate dehydrogenase |
| *fumC* | 3.5 | 4.0 | fumarase C, fumarate hydratase Class II |
| *aldA* | 2.0 | 2.2 | aldehyde dehydrogenase, NAD-linked |
| *fumB* | -18.5 | -23.5 | fumarase B, fumarate hydratase Class I |
| *frdB* | -10.7 | -11.8 | fumarate reductase, anaerobic, iron-sulfur protein subunit |
| *adhE* | -10.5 | -9.1 | CoA-linked acetaldehyde dehydrogenase and iron-dependent alcohol dehydrogenase |
| *hypB* | -9.1 | -10.5 | guanine-nucleotide binding protein, nickel donor for large subunit of hydrogenase 3 |
| *frdC* | -6.9 | -5.5 | fumarate reductase, membrane anchor polypeptide |
| *pykF* | -5.2 | -6.0 | pyruvate kinase I |
| *frdD* | -4.2 | -4.4 | fumarate reductase, membrane anchor polypeptide |
| *fhlA* | -3.7 | -3.5 | formate hydrogen-lyase transcriptional activator for fdhF |
| *hycB* | -3.3 | -4.0 | probable small subunit of hydrogenase-3, iron-sulfur protein |
| *fdhF* | -3.0 | -3.3 | selenopolypeptide subunit of formate dehydrogenase H |
| *pykA* | -2.7 | -2.7 | pyruvate kinase II, glucose stimulated |

Presented genes are derived from significant enrichment in the gene ontology fermentation. n=4
