# Supplementary material for: Global gene expression profiling and antibiotic susceptibility after repeated exposure to the carbon monoxide-releasing molecule-2 (CORM-2) in multidrug-resistant ESBL-producing uropathogenic Escherichia coli
Source: PLoS One. 2017 Jun 7;12(6):e0178541. doi: 10.1371/journal.pone.0178541 (PMC5462378; doi:10.1371/journal.pone.0178541)
Supplement: S3 Table — n = 4 (DOCX) [file pone.0178541.s003.docx]

**S3 Table**. **ESBL-producing *E. coli* genes associated with fimbriae and flagella that are differentially expressed following exposure to CORM-2 (250 µM) versus vehicle (2.5% DMSO).**

| **Gene** | **Fold change** | **Fold change** | **Gene product** |
| --- | --- | --- | --- |
| **symbol** | **First exposure** | **20x pre-exposed** |  |
|  | **CORM-2 vs** | **CORM-2 vs** |  |
|  | **first exposure** | **20x pre-exposed** |  |
|  | **vehicle** | **vehicle** |  |
| ECs211 | 6.5 | 9.3 | putative type 1 fimbrial protein precursor |
| *flhA* | 2.4 | 3.4 | putative flagellar export pore protein |
| *cheY* | -4.6 | -2.2 | chemotaxis regulator to flagellar motor component |
| *yehD* | -4.1 | -2.9 | putative fimbrial-like adhesin protein |

n=4
