# Supplementary material for: Global gene expression profiling and antibiotic susceptibility after repeated exposure to the carbon monoxide-releasing molecule-2 (CORM-2) in multidrug-resistant ESBL-producing uropathogenic Escherichia coli
Source: PLoS One. 2017 Jun 7;12(6):e0178541. doi: 10.1371/journal.pone.0178541 (PMC5462378; doi:10.1371/journal.pone.0178541)
Supplement: S4 Table — n = 4 (DOCX) [file pone.0178541.s004.docx]

**S4 Table**. **ESBL-producing *E. coli* genes associated with aerobic and anaerobic respiration that is differentially expressed following exposure to CORM-2 (250 µM) versus vehicle (2.5% DMSO).**

| **Gene** | **Fold change** | **Fold change** | **Gene product** |
| --- | --- | --- | --- |
| **symbol** | **First exposure** | **20x pre- exposed** |  |
|  | **CORM-2 vs** | **CORM-2 vs** |  |
|  | **first exposure** | **20x pre-exposed** |  |
|  | **vehicle** | **vehicle** |  |
| *ndh* | 11.1 | 10.6 | respiratory NADH dehydrogenase |
| *nuoF* | -7.8 | -7.3 | NADH:ubiquinone oxidoreductase, chain F |
| *nuoE* | -7.0 | -7.6 | NADH:ubiquinone oxidoreductase, chain E |
| *nuoA* | -6.9 | -6.7 | NADH:ubiquinone oxidoreductase, membrane subunit A |
| *torC* | -6.6 | -7.6 | cytochrome c-type subunit |
| *nuoC* | -6.3 | -7.0 | NADH:ubiquinone oxidoreductase, fused CD subunit |
| *torA* | -5.9 | -5.1 | trimethylamine N-oxide reductase subunit |
| *nuoB* | -5.5 | -5.5 | NADH:ubiquinone oxidoreductase, chain B |
| *nuoM* | -4.7 | -6.0 | NADH:ubiquinone oxidoreductase, membrane subunit M |
| *nuoH* | -4.5 | -5.1 | NADH:ubiquinone oxidoreductase, membrane subunit H |
| *nuoG* | -4.3 | -5,0 | NADH:ubiquinone oxidoreductase, chain G |
| *nuoI* | -4.1 | -4.8 | NADH:ubiquinone oxidoreductase, chain I |
| *torD* | -4.1 | -4.1 | part of trimethylamine-N-oxide oxidoreductase |
| *nuoJ* | -3.0 | -2.9 | NADH:ubiquinone oxidoreductase, membrane subunit |
| *nuoN* | -2.3 | -2.3 | NADH:ubiquinone oxidoreductase, membrane subunit N |

n=4
