# Supplementary material for: Global gene expression profiling and antibiotic susceptibility after repeated exposure to the carbon monoxide-releasing molecule-2 (CORM-2) in multidrug-resistant ESBL-producing uropathogenic Escherichia coli
Source: PLoS One. 2017 Jun 7;12(6):e0178541. doi: 10.1371/journal.pone.0178541 (PMC5462378; doi:10.1371/journal.pone.0178541)
Supplement: S5 Table — n = 4 (DOCX) [file pone.0178541.s005.docx]

**S5 Table**. **Differentially expressed genes that were not shared and present only after first-time exposure or only after pre-exposure 20 times to CORM-2 (250 µM).**

| **Gene symbol** | **Fold change** | **Gene product** |
| --- | --- | --- |
|  | **First exposure CORM-2 vs first exposure vehicle** |  |
| *ypeB* | 3.2 | predicted protein |
| *sppA* | 2.4 | protease IV, a signal peptide peptidase |
| *ubiG* | 2.4 | 3-demethylubiquinone-9 3-methyltransferase and 2-octaprenyl-6-hydroxy phenol methylase |
| *gcvA* | 2.4 | positive regulator of gcv operon |
| *trpR* | 2.3 | regulator for trp operon and aroH |
| *acrA* | 2.2 | acridine efflux pump |
| *yjeH* | 2.1 | putative transport |
| *speG* | 2.0 | spermidine N1-acetyltransferase |
| *dapB* | -3.0 | dihydrodipicolinate reductase |
| *slyD* | -2.9 | FKBP-type peptidyl-prolyl cis-trans isomerase |
| *fabG* | -2.4 | 3-oxoacyl-[acyl-carrier-protein] reductase |
| *pepD* | -2.4 | aminoacyl-histidine dipeptidase |
| *caiD* | -2.2 | carnitine racemase |
| *modA* | -2.1 | molybdate-binding periplasmic protein precursor |
|  | **20x pre-exposed CORM-2 vs 20x pre-exposed vehicle** |  |
| *tesA* | 2.3 | acyl-CoA thioesterase I |
| *agaR* | 2.2 | putative DEOR-type transcriptional regulator of aga operon |
| *csgC* | 2.2 | putative curli production protein |
| *arnF* | 2.1 | undecaprenyl phosphate-alpha-L-ara4N exporter |
| *yifK* | -2.4 | putative amino acid/amine transport protein |
| *yeiP* | -2.3 | putative elongation factor |
| *rplQ* | -2.2 | 50S ribosomal subunit protein L17 |
| *folA* | -2.0 | dihydrofolate reductase |
| *tam* | -2.0 | putative enzyme |
| *tuf* | -2.0 | protein chain elongation factor EF-Tu |

n=4
